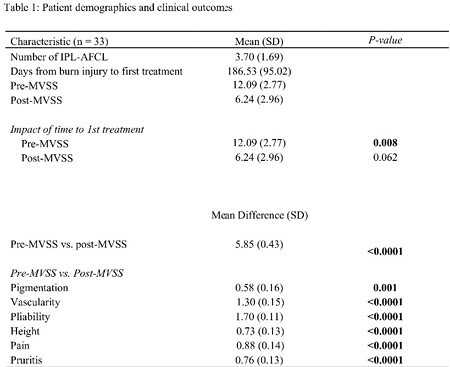# 81 Effectiveness of Simultaneous Intense Pulsed Light and Fractional CO2 Laser Therapy in Hypertrophic Burn Scars

**DOI:** 10.1093/jbcr/irae036.080

**Published:** 2024-04-17

**Authors:** Djoni Elkady, Brandon M Larson, Steffi Sharma, Richard B Lou, Anjay Khandelwal

**Affiliations:** Loyola University Chicago Stritch School of Medicine, North Olmsted, Ohio; Summa Health, Akron, Ohio; Akron Children's Hospital, Akron, Ohio; Loyola University Chicago Stritch School of Medicine, North Olmsted, Ohio; Summa Health, Akron, Ohio; Akron Children's Hospital, Akron, Ohio; Loyola University Chicago Stritch School of Medicine, North Olmsted, Ohio; Summa Health, Akron, Ohio; Akron Children's Hospital, Akron, Ohio; Loyola University Chicago Stritch School of Medicine, North Olmsted, Ohio; Summa Health, Akron, Ohio; Akron Children's Hospital, Akron, Ohio; Loyola University Chicago Stritch School of Medicine, North Olmsted, Ohio; Summa Health, Akron, Ohio; Akron Children's Hospital, Akron, Ohio

## Abstract

**Introduction:**

Hypertrophic burn scars present challenges for patients and clinicians. Intense Pulsed Light (IPL) and Ablative Fractional CO2 Lasers (AFCL) have shown promise in scar treatment by targeting vascular structures and promoting collagen production. While IPL-AFCL has been used in managing hypertrophic scarring in various dermatological conditions, limited data exist for burn scar treatment. In addition, scar vascularity can frequently limit the depth of penetration and density of AFCL yielding delayed results. The author's conducted a study assessing the efficacy of simultaneous IPL-AFCL therapy on hypertrophic burn scars using the Modified Vancouver Scar Scale (MVSS). To the author's knowledge, this is the first reported case series of simultaneous laser treatment in the treatment of hypertrophic burn scars.

**Methods:**

In this retrospective study (April 2021 to July 2023), data from patients (pediatric and adult) receiving at least two IPL-AFCL treatments were analyzed. Demographics, burn details, and complications were collected. Linear regression assessed the impact of time from burn to first treatment on MVSS scores. Unpaired t-tests compared pre/post-MVSS scores for partial-thickness (PT) and full-thickness (FT) burns, and paired t-tests evaluated pre/post-treatment MVSS score differences after IPL-AFCL treatment.

**Results:**

The study involving 33 patients (11 PT burns, 22 FT burns), the mean pre-MVSS score was 12.09 ± 2.77, and post-MVSS score was 6.24 ± 2.96. IPL-AFCL significantly reduced MVSS scores by a mean of 5.85 ± 0.43 (p < 0.0001) with an average of 3.70 ± 1.69 IPL-AFCL treatments per patient. Significant reductions were also observed in pigmentation (p=0.001), vascularity, pliability, height, pain, and pruritus (p < 0.0001). Linear regression analysis showed that the duration from burn injury to the first laser treatment significantly influenced pre-MVSS scores (p=0.008) but not post-MVSS (p=0.062). No significant differences were found in pre-MVSS (p=0.186) and post-MVSS (p=0.233) scores between PT and FT burns. As for complications, 4/33 patients experienced blistering following treatment which did not result in any long-term consequences.

**Conclusions:**

Our study demonstrated that IPL-AFCL treatment effectively improved hypertrophic burn scars. MVSS scores did not significantly differ between PT and FT burns, but the time from the initial burn to the first treatment significantly influenced pre-MVSS scores.

**Applicability of Research to Practice:**

Simultaneous use of different light/laser modalities is safe and effective and may offer advantages with symptomatic, functional and cosmetic benefits.